# Microbiological diagnosis of endophthalmitis using nanopore targeted sequencing

**DOI:** 10.1111/ceo.13992

**Published:** 2021-09-15

**Authors:** Qiong Huang, Aisi Fu, Yiyan Wang, Jie Zhang, Wanxu Zhao, Yang Cheng

**Affiliations:** ^1^ Department of Ophthalmology, Union Hospital, Tongji Medical College Huazhong University of Science and Technology Wuhan China; ^2^ Key Laboratory of Combinatorial Biosynthesis and Drug Discovery Ministry of Education and Wuhan University School of Pharmaceutical Sciences Wuhan China; ^3^ Wuhan Dgensee Clinical Laboratory Co., Ltd Wuhan China

**Keywords:** culture, endophthalmitis, false‐negative, nanopore targeted sequencing

## Abstract

**Background:**

Microorganism identification is critical for the early diagnosis and management of infectious endophthalmitis, but traditional culture can yield false‐negative results. Nanopore targeted sequencing (NTS) is a third‐generation sequencing technique with multiple advantages. This study aimed to test aqueous humour or vitreous fluid samples from presumed cases of infectious endophthalmitis using NTS to evaluate the feasibility of NTS in diagnosing endophthalmitis, especially for culture‐negative cases.

**Methods:**

This prospective study enrolled patients who presented to the Department of Ophthalmology of Union Hospital (Wuhan, China) between June 2018 and December 2020. The samples were sent immediately for routine microbiology culture processing and NTS assay.

**Results:**

NTS identified microorganisms in 17 of 18 cases (94.4%) (eight culture‐positive cases, nine culture‐negative cases, and one case unavailable for culture). There was a high‐quality match between culture and NTS for culture‐positive cases. In the eight culture‐negative cases and the case unavailable for culture, NTS detected either bacteria, fungi, or a mixture of bacteria and fungi in the intraocular fluids. The average waiting times for the results of bacterial and fungal cultures were 48 and 72 h, respectively. The average time for the NTS results was 12 h.

**Conclusions:**

NTS appears to be a promising diagnostic platform for diagnosing infectious endophthalmitis, even for culture‐negative cases.

## 1 INTRODUCTION

Endophthalmitis is an intraocular infection caused by bacteria or fungi involving the vitreous and/or aqueous humour. Severe or irreversible vision loss can occur within hours or days of symptom onset. Therefore, a prompt diagnosis and treatment are necessary to preserve vision.^1^, ^2^ Endophthalmitis is rare, and most cases occur after ocular surgery, injections or trauma (exogenous infection).^2^ Cataract surgery is the most common cause of endophthalmitis, happening in 0.063%–0.195% of the patients.^3^ Other procedures such as vitrectomy, keratoplasty, trabeculectomy or glaucoma drainage implant insertion can cause endophthalmitis as well.^2^, ^4^, ^5^ Ocular injections, keratitis and penetrating eye trauma can also be the source of infection.^2^, ^4^, ^5^ Endogenous endophthalmitis is caused by the hematogenous spread of infection to the eye and is a rare complication in patients with bacteremia or fungemia.^2^, ^4^, ^5^ The most common pathogens vary depending on the type of infection (exogenous or endogenous) and causative events (surgery, ocular injection or trauma).^2^, ^4^, ^5^

The identification and characterisation of the causal microorganism from routine culture are limited and of low sensitivity owing to multiple factors, and negative culture results cannot rule out the diagnosis in clinically suspected infectious endophthalmitis.^2^, ^4^, ^5^ Research on alternative molecular diagnostic methods has produced various strategies that improve microorganism detection.^6^, ^7^ Recent genomic sequencing techniques allow the highly sensitive detection of any microorganism in a specimen. The sequenced tags are compared against a large database generated from the NCBI Genbank, containing all known DNA sequences, and the database is updated daily to incorporate the most current available data and can identify tags from mammalian, bacterial, fungal, parasitic and viral organisms.^8^, ^9^ Deep sequencing techniques have been widely used to detect microorganisms in diagnosing infectious encephalitis, meningitis, endocarditis, liver abscess and endophthalmitis independent of culture.^10^, ^11^, ^12^, ^13^, ^14^, ^15^, ^16^ These techniques offer the promise of improving the detection of traditional microorganisms and identifying microorganisms not previously associated with endophthalmitis, and being more likely to become a routine diagnostic tool in ocular microbiological laboratories.

The nanopore sequencer is amongst the third‐generation sequencing platforms, and it identifies the DNA sequences from the change in electrical current resulting from a DNA strand being forced through a nanometre‐sized pore embedded in a membrane.^17^, ^18^ This method can achieve DNA sequencing while maintaining low cost, high accuracy, long read length and high throughput.^16^, ^19^, ^20^

Therefore, this study aimed to test aqueous humour or vitreous fluid samples from presumed infectious endophthalmitis using nanopore targeted sequencing (NTS) to evaluate the feasibility of NTS in diagnosing endophthalmitis, especially for culture‐negative cases.

## 2 METHODS

### 2.1 Study design and participants

This prospective study enrolled patients who presented to the Department of Ophthalmology of Union Hospital (Wuhan, China) between June 2018 and Dec 2020. The study was approved by the ethics committee of Union Hospital ([2020]0609–01) and adhered to the tenets of the Declaration of Helsinki. The participants provided written informed consent.

All patients were clinically presumed with infectious endophthalmitis and underwent anterior chamber wash/vitreous tap or pars plana vitrectomy (PPV). Presumed infectious endophthalmitis was defined as inflammation caused by a suspected bacterial or fungal infection.^2^, ^21^, ^22^ The symptoms included sudden vision loss, ocular pain, photophobia, red eye, anterior chamber cells and flare, hypopyon, vitreous cell clumping, and vitreous opacity. There were no exclusion criteria.

### 2.2 Data collection

Demographic data and clinical characteristics were collected, including age, sex, predisposing factors (including underlying disease, history of eye disease, history of ocular surgery, history of ocular trauma and history of bacteria or fungal infection from other sources), initial visual acuity and duration of clinical symptoms.

### 2.3 Specimens

Aqueous humour or vitreous fluid specimens were collected (at least 50 μl) by three methods: anterior chamber wash, vitreous tap and PPV. Aqueous humour was collected when anterior chamber irrigation and aspiration were performed. A vitreous tap was performed using 1‐ml syringes and 29‐G needles by aspiration after insertion into the vitreous cavity to a depth of at least 1 cm. A 23‐G or 25‐G PPV was performed by retina surgeons. Following the placement of the vitrectomy ports, the vitreous fluid was aspirated using a vitrectomy cutter while the infusion system was turned off shortly or under air infusion to maintain the pressure in the eye.

### 2.4 Culture

The aqueous humour or vitreous fluid specimens were sent immediately for routine microbiology culture processing and NTS assay. The specimens were first inoculated in Columbia blood agar base medium for bacteria and in Sabouraud dextrose agar medium for fungi, using a BACTEC 9120 culture system (BD Diagnostics, Sparks, MD). In cases with positive cultures, the isolated fungi or/and bacteria were identified using a Vitek 2 Compact automated identification system (bioMerieux, Marcy l'Étoile, France) and a MALDI Biotyper mass analyser (Bruker, Madison, WI).

### 2.5 Nanopore targeted sequencing

**FIGURE 1:**
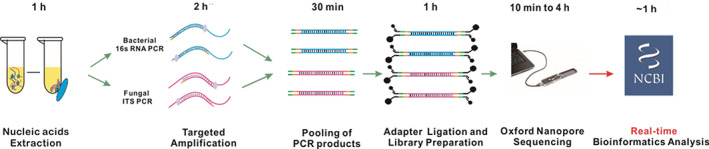
Nanopore targeted sequencing detection

NTS was conducted according to the method described by Wang et al., as shown in Figure 1.^23^ DNA was extracted using the QIAamp UCP Pathogen Mini Kit (Qiagen, Venlo, The Netherlands). The barcoded products of the 16 s rRNA, ITS1/2 and rpoB genes from the same samples were amplified and pooled according to a mass ratio of 10:3:1, as previously described.^23^ The pooled products from the different samples were mixed equally and used to construct sequencing libraries using the 1D Ligation Kit (SQK‐LSK109; Oxford Nanopore Technologies, Oxford, UK). The library was sequenced using a MinION or GridION system (Oxford Nanopore Technologies, Oxford, UK). The TE buffer was assayed in each batch as a negative control. The samples were tested positive for bacteria or fungi if they met any of the established thresholds after bioinformatic analysis. The detected microorganisms were described as critical pathogens, opportunistic pathogens or typically nonpathogenic commensal microbes according to the published literature and clinical guidelines.^2^, ^22^

### 2.6 Treatment and follow‐up

Treatment plans were selected based on the initially presumed type of infection and adjusted according to the NTS and/or culture results. All participants were scheduled for follow‐up visits at 1 week, 1 month and 3 months. Follow‐up examination data were recorded at each visit, including visual acuity, intraocular pressure, infection evolution and anterior segment/fundus assessment.

### 2.7 Statistical analysis

Only descriptive statistics were used. The results from the two identification methods were compared.

## 3 RESULTS

### 3.1 Participants

Eighteen patients with clinically presumed infectious endophthalmitis were enrolled. The clinical and demographic characteristics of the patients are presented in Table 1. There were four females and 14 males. The participants were 50.5 ± 14.5 years of age. The most probable factors causing the presumed infectious endophthalmitis were an endogenous source in eight (44.4%) participants, a trauma in six (33.3%) and cataract surgery or vitrectomy in four (22.2%). Patient #6 had a history of liver abscess. Past culture for the liver abscess reported *Klebsiella pneumoniae* infection. Patient #16 had a history of heart transplantation and was diagnosed with a lung infection.

**TABLE 1:** Clinical and demographic details of the patients with presumed infectious endophthalmitis

Sample ID	Age^a^	Sex	Diagnosis	Predisposing factor	Duration	Initial VA	Surgery	Treatment	Final VA
1	70s	F	Exogenous endophthalmitis	Injury: wood	36 h	LP	PPL + PPV + silicone Oil	V + C	HM
2	20s	M	Exogenous endophthalmitis	Injury: bamboo pole in a pigpen	5 h	CF	PPL + PPV + silicone Oil	V + C	20/400
3	40s	M	Endogenous endophthalmitis	–	15 days	HM	PPV + silicone Oil	V + C	20/40
4	50s	M	Bilateral endogenous endophthalmitis	–	11 days	OS: HM, OD: 20/200	OU:PPV+ silicone Oil	Amp‐B	OU:CF
5	50s	F	Endogenous endophthalmitis	–	30 days	20/200	PPV	Amp‐B	20/80
6	60s	M	Endogenous endophthalmitis	Liver abscess	15 days	HM	PPV + silicone Oil	V + C	FC
7	30s	M	Endogenous endophthalmitis	Chemotherapy for liver cholangiocarcinoma	15 days	LP	PPL + PPV + silicone oil	V + C +Amp‐B	Evisceration
8	50s	M	Endogenous endophthalmitis	Diabetes, liver abscess	3 days	LP	PPL + PPV	V + C	Evisceration
9	50s	M	Traumatic endophthalmitis	Injury: intraocular foreign body, traumatic cataract surgery	3 days	LP	2 PPVs	V + C, Amp‐B, multi voriconazole	NLP, atrophia bulbi
10	20s	M	Traumatic endophthalmitis	Intraocular foreign body (iron wire)	3 days	HM	PPL + PPV+ IOFB‐R + silicone oil	V + C	CF
11	30s	M	Endogenousendophthalmitis	Post removal of urinary calculi surgery	15 days	20/100	PPV	Voriconazole	20/40
12	60s	M	Postoperative endophthalmitis	Cataract surgery: 15 days	15 days	HM	PPV + AC Wash	V + C	20/200
13	50s	M	Exogenous endophthalmitis	Intraocular foreign body perforation	37 h	LP	PPL + PPV + IOFB‐R + silicone oil	V + C	CF
14	30s	M	Postoperative endophthalmitis	Cataract surgery + PPV + silicone oil (for traumatic vitreous haemorrhage)‐1 day after intraorbital foreign body removal: 7 days	1 day	LP	AC wash	V + C	CF
15	70s	F	Postoperative endophthalmitis	Cataract surgery: 10 days, diabetes	10 days	LP	PPV + silicone oil	V + C; Amp‐B	CF
16	50s	F	Endogenous endophthalmitis	Heart transplantation: 17 days, respiratory infection, diabetes	NA	LP	PPL + PPV,2nd PPV	Multi‐voriconazole, multi‐Amp‐B	NLP atrophia bulbi
17	30s	M	Exogenous endophthalmitis	Perforation: steel wire	1 day	LP	PPL + PPV, 2nd silicone oil tamponade	V + C	20/400
18	60s	M	Postoperative endophthalmitis	Cataract surgery: 4 days	4 days	20/100	PPV	V + C	20/20

Abbreviations: AC wash, anterior chamber wash; Amp B, amphotericin B; C, ceftazidime; CF, counting fingers; F, female; HM, hand movements; IOFB‐R, intraocular foreign body removal; LP, light perception; M, male; NPL, no light perception; PPL, pars plana lensectomy; PPV, pars plana vitrectomy; V, vancomycin; VA, visual acuity.

a For anonymity, the ages of included patients were presented in terms of approximate range.

### 3.2 Treatment

**FIGURE 2:**
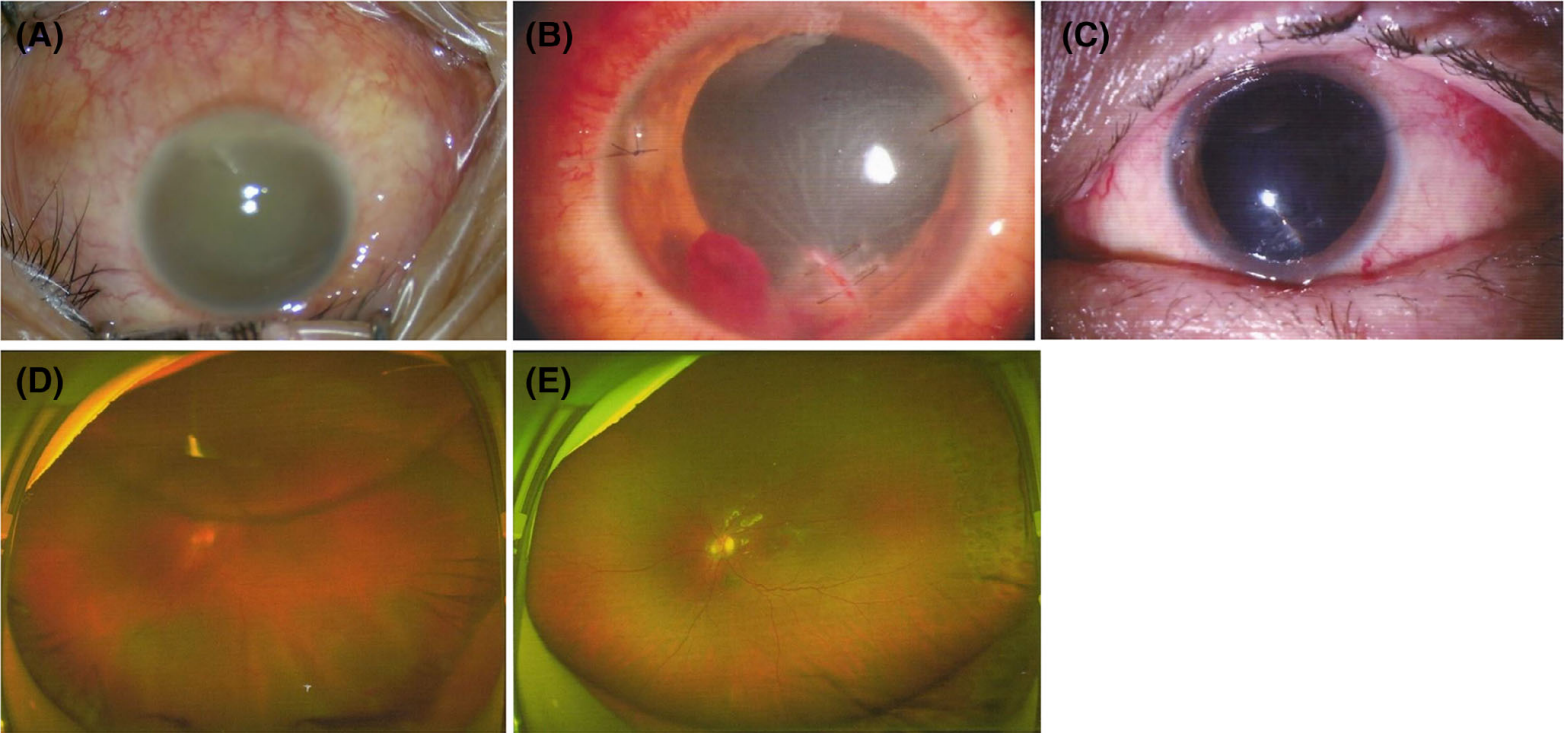
A man in his 30s (case #17) presented to the hospital emergency department complaining of redness and pain in his left eye with blurred vision. His left eye was performed by a steel wire 1 day before. The visual acuity on the left eye was only light perception. (A) Upon examination, an inferior corneal wound and edema were detected, and hypopyon and lens opacity developed. (B) He was diagnosed with bacterial endophthalmitis and admitted. Emergent corneal suturing, lensectomy, and vitrectomy were performed immediately. Empirical intravitreal injection of antibiotics (ceftazidime, 2.25 mg/0.1 ml; vancomycin, 1 mg/0.1 ml) was also performed. He was prescribed topical levoflocaxin (0.3%) and tobradex (tobramycin 0.3% and dexamethasone 0.1%). NTS and culture results of the anterior humour and vitreous fluid showed *Bacillus cereus*. (C) The infection subsided 1 week later. (D) Retinal detachment developed 3 weeks after the surgery, and the corrected visual acuity was 0.1. (E) Silicone oil tamponade was performed, and the retina was reattached. NTS, nanopore targeted sequencing

For the participants with suspected bacterial infection, an initial injection of intravitreal antibiotics (vancomycin 1 mg/0.1 ml and ceftazidime 2.25 mg/0.1 ml) was performed in two participants, in addition to PPV in three participants, and PPV combined with lensectomy in five. PPV was also performed on patient #6, who was clinically diagnosed with bacterial endophthalmitis and accompanying liver abscess, although with negative results on the later NTS assay and culture. There was a clinical suspicion of fungal infection in four patients, and additional intravitreal amphotericin B (10 μg/0.1 ml) or voriconazole (100 μg/0.1 ml) was given, including three patients who underwent PPV and one who underwent PPV combined with lensectomy. According to NTS and culture results, there was a conversion of agents in one patient (#9), who was initially presumed to be a bacterial infection and was finally identified as a fungal infection. A second vitrectomy and multiple intravitreal voriconazole or amphotericin‐B injections were performed in two participants with fungal endophthalmitis who later developed atrophia bulbi with no light perception. Two patients initially presumed to be with bacterial infection underwent anti‐fungal agent intravitreal injection when NTS revealed a mixture of bacterial and fungal infection. The treatments included topical levofloxacin (0.3%) or moxifloxacin (0.5%), Tobradex (tobramycin 0.3% and dexamethasone 0.1%) and intravenous sensitive anti‐bacterial or anti‐fungal (voriconazole) agents. Fourteen out of the 18 patients had improved visual acuity after treatment, two patients could keep their eyeballs, and two patients finally had to undergo evisceration. The examination and treatment of one patient (#17) are presented in Figure 2.

### 3.3 Microbiology culture

Seventeen samples were sent for microbiology culture (the sample from one participant was not available for microbiology culture). The culture‐positive rate was 47.1% (8/17), and seven participants were positive for bacteria, including group G streptococcus, *Streptococcus viridans*, *Bacillus cereus*, *K. pneumoniae*, *Staphylococcus epidermidis* and Pan bacteria (e. agglomerans), and one participant was positive for *Aspergillus fumigatus* (Table 2). Nine out of the 17 cultured samples were negative by microbiology culture. The average waiting times for the results of bacterial and fungal cultures were 48 and 72 h, respectively.

**TABLE 2:** Taxonomic lineage identified in culture‐positive cases of endophthalmitis

Sample ID	Microbiology culture	DNA sequencing
1	Group G *streptococcus*	*Streptococcus* (95%)*, Staphylococcus* (5%)
2	*Streptococcus viridans*, *Bacillus cereus*	*Streptococcus suis, Clostridium perfringens*
8	*Klebsiella pneumoniae*	*K. pneumoniae*
9	*Aspergillus fumigatus*	*Aspergillus fumigatus*
12	*Staphylococcus epidermidis*	*S. epidermidis*
13	Pan bacteria (e. agglomerans)	*Pantoea ananatis*
17	*B. cereus*	*B. cereus*
18	*S. epidermidis*	*S. epidermidis* (79.3%), *Finegoldia magna* (20.7%)*, Candida parapsilosis*

### 3.4 Nanopore targeted sequencing

NTS detected the presence of microorganisms in 17 samples out of 18 participants (positive rate of 94.4%), including the eight culture‐positive patients, eight culture‐negative patients and the patient with no specimen for culture (Table 2). Neither culture nor NTS detected any microorganisms in the specimen of patient #6. Amongst the eight culture‐positive specimens, NTS detected bacteria in six patients, fungus in one, and a mixture of bacteria and fungus in one patient. Amongst the eight culture‐negative specimens and the case that was not available for culture, NTS detected bacteria in three cases, fungi in four, and a mixture of bacteria and fungi in two, while showing negative results in case #6. In addition, NTS showed multiple microbial organisms in five of the 17 participants, amongst which three were a mixture of bacteria and fungi. Of note, NTS even detected *Streptococcus suis* in case #2, which concur with the history of eyeball perforation by a bamboo pole from a pigpen. The average time for the NTS confirmation was 12 h.

### 3.5 Concordance between culture and NTS for culture‐positive endophthalmitis

There was good agreement between culture and NTS results for the culture‐positive patients, as shown in Table 2. All eight culture‐positive patients showed the presence of DNA of the same microorganism by NTS or the simultaneous presence of other species of bacteria or a mixture of bacteria and fungi. In five patients, the bacteria that grew in culture were also identified by NTS assay as a mono‐microorganism infection, including *K. pneumoniae*, *S. epidermidis*, Pan bacteria (e. agglomerans), *B. cereus* and *Aspergillus fumigatus*. Patient #1 showed group G *Streptococcus* in culture, but NTS showed more accurate data on the sample having predominantly DNA of *Streptococcus* (95%) and *Staphylococcus* (5%). Patient #2 showed *Streptococcus viridans* and *B. cereus* in culture, while NTS showed DNA of *S. suis* and *Clostridium perfringens*, which was more concordant with the history of eyeball perforation by a bamboo pole from a pigpen. Patient #18 showed *S. epidermidis* in culture, while NTS showed a mixture of *S. epidermidis* (79.3%), *Finegoldia magna* (20.7%) and *Candida parapsilosis*.

### 3.6 NTS for culture‐negative endophthalmitis or cases not available for culture

In eight culture‐negative patients and the case that was not available for culture, NTS detected either bacteria, fungi, or a mixture of bacteria and fungi in the intraocular fluid specimens (Table 3). NTS did not detect any microorganism in patient #6, nor did the culture, which might be due to microorganism degradation with previous long‐term intravenous anti‐bacterial agents treatment before referral to the eye clinic. Amongst the eight culture‐negative patients, NTS showed three patients with bacteria (*Pseudomonas aeruginosa*, *S. epidermidis* and a mixture of 88.3% *Ralstonia insidiosa* and 11.7% *S. epidermidis*), three patients were with one fungus (*Aspergillus fumigatus* and *Candida albicans*), and two cases had a mixture of bacteria and fungi (a mixture of *K. pneumoniae* and *Cutaneotrichosporon arboriformis*, a mixture of *S. epidermidis* and *C. albicans*). In the case that was not available for culture, NTS detected *Aspergillus fumigatus* in the vitreous.

**TABLE 3:** Taxonomic lineage identified in cases with a culture‐negative result or unavailable for culture

Sample ID	Microbiology culture	DNA sequencing
3	–	*Pseudomonas aeruginosa*
4	–	*Aspergillus*
5	–	*Aspergillus*
6	–	*–*
7	–	*Klebsiella pneumoniae, Cutaneotrichosporon arboriformis*
10	–	*Staphylococcus epidermidis*
11	–	*Candida albicans*
14	–	*Ralstonia insidiosa* (88.3%), *S. epidermidis* (11.7%)
15	–	*S. epidermidis*, *C. albicans*
16	NA	*Aspergillus fumigatus*

Abbreviation: NA, not applicable.

## 4 DISCUSSION

Microorganism identification is critical for the early diagnosis and management of infectious endophthalmitis, but traditional culture can yield false‐negative results.^2^, ^4^, ^5^ NTS is a third‐generation sequencing technique with multiple advantages.^16^, ^19^, ^20^ This study aimed to test aqueous humour or vitreous fluid samples from patients with presumed infectious endophthalmitis using nanopore sequencing to evaluate its feasibility in diagnosing endophthalmitis, especially for culture‐negative cases. The results suggest that NTS might be a promising diagnostic platform for diagnosing infectious endophthalmitis, even for culture‐negative cases.

Endophthalmitis, either exogenous or endogenous, is one of the most devastating eye conditions and can lead to irreversible blindness in the infected eye within hours or days of symptom onset.^2^, ^4^, ^5^ The diagnosis of endophthalmitis depends mostly on the clinical findings during an ophthalmological examination.^2^, ^4^, ^5^ Compared with exogenous endophthalmitis characterised by the presence of ocular trauma or ocular surgery history, endogenous endophthalmitis might be nonspecific and masquerading as panuveitis, leading to misdiagnosis or delayed diagnosis,^1^, ^2^ with a high risk of visual loss or eyeball loss, but also increased risk of mortality.^24^, ^25^ Early diagnosis depends not only on the ophthalmologists' high alertness but is also supported by microorganism detection of intraocular fluid in presumed endophthalmitis.

Despite the inability to detect certain microorganisms and relatively low yield, microbiology culture is the current gold standard for the diagnosis of most intraocular infections. Negative cultures do not rule out the diagnosis since 50%–60% of endophthalmitis cases are culture‐negative,^2^, ^4^, ^5^ which is consistent with the present study. In addition, the waiting time for culture results can vary from 2 to 12 days, and the lack or delay of microbiological culture confirmation can lead to the inappropriate use of some treatments.

Molecular diagnostic techniques can reveal microorganisms in many culture‐negative samples, and these techniques play a larger role in endophthalmitis diagnosis.^10^, ^11^, ^12^, ^13^, ^14^, ^15^, ^16^ PCR has shown promise for the management of endophthalmitis since its first application in ophthalmology in 1993 and has improved the yield of detection and reduced the time to make a confirmatory diagnosis.^6^, ^26^, ^27^ Nevertheless, the number of fungi and/or bacteria that can be simultaneously detected is limited due to differences in amplification efficiencies of different primer sets and the limited number of fluorescent labels.^6^, ^26^, ^27^

In comparison, genomic sequencing does not target just one specific species but can detect all the different microorganisms present in a clinical sample in one single assay. Whole‐genome high‐throughput sequencing of microbial communities (metagenomics) has revolutionised microbial ecology, clinical microbiology and industrial biotechnologies. This technique allows improved detection of traditional microorganisms and can identify microorganisms not previously associated with endophthalmitis.^8^, ^9^ Genomic sequencing shows superiority for identifying the pathogens responsible for ocular infections, with the potential to improve the accuracy and speed of diagnosis and hastening the selection of the optimal therapy. Researchers from India showed that next‐generation sequencing is a good tool for microbial research in endophthalmitis in 34 cases of presumed infectious endophthalmitis, and an extension study in 75 cases confirmed the feasibility of high‐throughput sequencing in an ocular clinical setting for the diagnosis of infectious endophthalmitis, especially in culture‐negative cases.^14^, ^15^

Since the release of MinION (Oxford Nanopore Technologies), the portability, affordability and speed of results of NTS make it suitable for real‐time applications, generating much excitement and interest in the genomics community. This specific method is considered desirable because it can achieve DNA sequencing while maintaining low cost, high accuracy, long read length and high throughput. Studies demonstrated the utility of nanopore sequencing, suggesting it as one of the most promising sequencing approaches.^16^, ^19^, ^20^ Here we report the outcome of a proof‐of‐concept study that used NTS to identify bacteria and fungi in patients with presumed infectious endophthalmitis. We found good concordance between NTS and culture for culture‐positive cases, as supported by previous reports comparing next‐generation sequencing and culture.^6^, ^9^, ^28^ The difference of sensitivity between NTS and culture is mainly owed to the theory that NTS is entirely based on microorganism DNA detection, while culture detects the presence of viable microorganisms and is dependent upon the actual number of pathogen cells in the specimen, the presence of fastidious microorganisms, and the use of antibiotics before specimen collection that may inhibit microbial growth during culture, or infection with non‐bacterial pathogens.^29^ In this study, timesaving NTS helped make the correct diagnosis and change the treatment in patient #9.

Similar to previous studies,^14^, ^15^, ^30^, ^31^ we detected more than one microorganism in some of the patients, and some of these microorganisms did not grow in culture (patients #1 and #18). Competition amongst microorganisms, fastidious culture, differences in growth rate, and quorum sensing are some of the plausible reasons. The NTS assay in this study helped detect a mix of bacteria and fungi in some patients (#7, #15 and #18), which was completely missed by culture.

The lack of identification of drug sensitivity is a major drawback of NTS. Still, clinically, the pathogens that cause endophthalmitis are mainly divided into three categories. For gram‐positive bacteria, vancomycin can be used empirically, while ceftazidime is used empirically for gram‐negative bacteria, and amphotericin B or voriconazole is used for fungi. Compared with regular culture, NTS can detect the pathogens more quickly, and the corresponding empirical medication can be started pending the culture and drug sensitivity results.

This study has limitations. The number of patients was small, and they were from a single centre. The diagnostic value of NTS could not be assessed using the conventional metrics (sensitivity, specificity, etc.) because of the small number of patients. Because of the small number of patients in the different diagnostic or treatment categories, only descriptive statistics could be used. Except for patients #6 and #16, patients with endogenous endophthalmitis were diagnosed based on positive intraocular pathogen detection in the absence of eye trauma, but no systemic cultures were performed. Further studies with larger sample size are needed to address mentioned question.

In conclusion, this study provides proof of concept that NTS is a powerful approach to identify the microorganisms in the intraocular fluid of patients with suspected infectious endophthalmitis, which is not only complementary but also superior to microbiological culture‐based approaches, especially considering the waiting time for laboratory result confirmation. It also has the potential to revolutionise the diagnosis of culture‐negative endophthalmitis, which is of great clinical significance for early diagnosis and facilitating prompt initiation of appropriate treatments.

## CONFLICT OF INTEREST

The author declares that there is no conflict of interest that could be perceived as prejudicing the impartiality of the research reported.
